# Retrospective Analysis of Doses Delivered during Embolization Procedures over the Last 10 Years

**DOI:** 10.3390/jpm12101701

**Published:** 2022-10-12

**Authors:** Joël Greffier, Djamel Dabli, Tarek Kammoun, Jean Goupil, Laure Berny, Ghizlane Touimi Benjelloun, Jean-Paul Beregi, Julien Frandon

**Affiliations:** 1 IMAGINE UR UM 103, Department of Medical Imaging, Montpellier University, Nîmes University Hospital, 30029 Nîmes, France; 2 Department of Medical Physics, Nîmes University Hospital, 30029 Nîmes, France

**Keywords:** embolization, dose optimization, interventional radiology, CT scan

## Abstract

Background: This study aimed to retrospectively analyze dosimetric indicators recorded since 2012 for thoracic, abdominal or pelvic embolizations to evaluate the contribution of new tools and technologies in dose reduction. Methods: Dosimetric indicators (dose area product (DAP) and air kerma (AK)) from 1449 embolizations were retrospectively reviewed from August 2012 to March 2022. A total of 1089 embolizations were performed in an older fixed C-Arm system (A1), 222 in a newer fixed C-Arm system (A2) and 138 in a 4DCT system (A3). The embolization procedures were gathered to compare A1, A2 and A3. Results: DAP were significantly lower with A2 compared to A1 for all procedures (median −50% ± 5%, *p* < 0.05), except for uterine elective embolizations and gonadal vein embolization. The DAP values were significantly lower with A3 than with A1 (*p* < 0.001). CT scan was used for guidance in 90% of embolization procedures. Conclusions: The last C-Arm technology allowed a median reduction of 50% of the X-ray dose. The implementation of a CT scan inside the IR room allowed for more precise 3D-guidance with no increase of the dose delivered.

## 1. Introduction

Thoracic, abdominal or pelvic embolizations represent an important proportion of all procedures performed in interventional radiology (IR), with many indications [1,2,3,4]. Embolizations may be long and complex procedures, with high radiation doses delivered to the patients, and it is possible that the patient skin dose may exceed the threshold of deterministic effects (2 to 3 Gy), leading to radiodermatitis or alopecia [5,6,7,8]. Another issue, even when relatively low doses are delivered in IR, is the risk of long-term stochastic effects, including induced cancer [9,10]. Optimization of IR practices is thus needed to reduce these risks [11].

The International Commission on Radiological Protection (ICRP) has recommended the collection and monitoring of dose indicators for each patient exposed to ionizing radiation [11]. In IR, this collection allows for improved detection and follow-up of patients at risk of a deterministic effect to improve their therapeutic management. In addition, an evaluation of the skin dose can be performed when the air kerma at the interventional reference point (AK) or the dose area product (DAP) are high [12,13].

To reduce the doses delivered during interventional procedures, the optimization principle must be applied with great rigor. Dose optimization consists in reducing radiation doses as low as reasonably achievable while maintaining sufficient image quality. For this purpose, the protocols are often optimized by the medical physicists and the resulting image quality for each protocol is validated by the interventional radiologists [14]. Medical physicists may also train radiologists on good patient radiation protection practices. Finally, manufacturers develop new equipment to reduce the dose delivered to the patients, with a sufficient image quality, using new tools or new modalities to facilitate guidance and improve patient management [15,16,17,18,19,20,21].

In our institution, dosimetric indicators have been collected daily by medical physicists for all IR procedures since 2012 and methods to calculate or measure the skin dose have been implemented since 2013 [22,23]. Between 2012 and 2020, all embolizations were performed in a single vascular room equipped with a fixed C-arm. In 2020, this room was replaced by two new IR rooms: a room with a fixed C-arm and equipped with the ClarityIQ technology, which allows reducing the dose while maintaining an equivalent image quality, and a second room with a fixed C-arm coupled with a CT-scan. The use of the CT-scan in this new room allows improving percutaneous and vascular guidance, controlling the environment of the treated area and performing a control at the end of the procedure. For these two new equipped rooms, the protocols and practices have also been optimized. We therefore assumed that the new features introduced in these two rooms may modify the doses delivered and the management of the patients.

The purpose of our study was to retrospectively analyze the dosimetric indicators recorded since 2012 and evaluate the contribution of the new tools and new rooms in dose reduction and patient management.

## 2. Materials and Methods

### 2.1. Patient Study

The present retrospective study was approved by the local institutional review board (Interface Recherche Bioethique Institutional Review Board, number 22.04.03) and patient approval was waived due to the study retrospective character. The study was carried out in accordance with current guidelines and regulations. Patients (or their legal guardians) were systematically informed that their data were collected in an anonymous manner for a retrospective study and that they could refuse at any time to participate in the study (non-opposition statement).

Data were acquired consecutively for all adult participants undergoing thoracic or abdomen-pelvic embolization from August 2012 to March 2022 in our institute. Ten embolization procedures were studied (Table 1):

- Bronchial artery embolizations (BE);
- Abdominal elective embolizations for scheduled treatment and visceral aneurysm (except for renal artery) treatments (AEE);
- Abdominal urgent embolizations for active bleedings or vascular injuries of digestive arteries (AUE);
- Hepatic chemoembolizations (HCE);
- Radioembolization for primary and metastatic liver cancers (RaE);
- Renal artery embolizations (RE);
- Pelvic embolizations for planned prostatic embolizations (PE);
- Uterine elective embolizations for leiomyomas or vascular malformations (UEE);
- Uterine urgent embolizations for postpartum hemorrhages (UUE);
- Gonadal vein embolizations (GVE).

Patients were not included in the study if they were under 18 years old or if they refused to participate in the study (opposition statement).

For all patients, the age, total dose area product (DAP), air kerma (AK), total fluoroscopy time (FT) and number of fluorography images were collected. The total dose length product (DLP) was collected only for patients who had undergone a CT scan during the procedure. For all procedures, the dosimetric indicators were collected daily from the dose reports available in the Picture Archiving and Communication System (PACS) or in the Dose Archiving and Communication System (DACS) by the medical physicists and were archived in a database.

### 2.2. X-ray Sources

From August 2012 to September 2020, all embolization procedures were performed on the fixed C arm Allura^®^ Xper FD 20 (Philips Healthcare Systems, Best, The Netherlands) system (A1). Since Sept ember 2020, the embolization procedures were performed on the fixed C-arm Azurion 7 M20 (Philips Healthcare Systems, Best, The Netherlands) system (A2) or on the Alphenix 4DCT (Canon Medical Systems, Otawara, Japan) system (A3), which combines a flat-panel Angio C-arm (Alphenix) and a CT unit (Aquilion One Genesis). Seven embolization procedures studied were preferentially performed using the A2 system (BE, AEE, AUE, RE, UEE, UUE and GVE) and the three others preferentially using the A3 system (HCE, PE and RaE).

For the two Philips systems, the pulsed fluoroscopy mode (7.5 pulses) with an additional filtration of 0.9 mm Cu and 0.1 mm for the Al system were used. Digital subtraction angiography images were used for all embolization procedures with a frame rate of 2 or 3 frames according to the procedure performed and an additional filtration of 0.1 mm Cu and 0.1 mm for the Al system. Cone-beam CT acquisition was used for all PEs but not for the other procedures.

For the C-arm of the 4DCT, the low-pulsed fluoroscopy mode (5 or 7.5 pulses) and the additional filtration were used (from 0.2 to 0.5 mm Cu) depending on the procedure type, the difficulties encountered and the operator. For all embolization procedures, digital subtraction angiography images were used at a 3-frames rate and an additional 0.2 mm-Cu filtration. CT acquisitions were usually performed during the procedure to assess the proper vascular targeting of the embolization. CT acquisitions were initially performed for planning, guidance or post-embolization control [15,22].

For the A1 and A2 systems, the detector was rectangular with a diagonal length of 48 cm while in A3, the diagonal length was 40 cm. Eight electronic zooms were available in A1 and A2 (diagonals of 48/42/37/31/27/22/19/15 cm) and six in A3 (diagonals of 40/30/20/15/11/8 cm). 

For the A1 and A2 systems, the AK was measured in air at a distance of 66 cm from the X-ray tube. For A3, the AK was measured in air at 55 cm from the X-ray tube but took into account the table and mattress attenuation. To compare the AK of the different systems, for A3, AK was corrected to be obtained at a 66-cm distance from the X-ray tube (correction factor of 0.694) and its measurement was performed in the air without taking into account the table and mattress attenuation (correction factor of 1.225).

The average field size during each procedure was calculated as the ratio of the total DAP to the total AK (in air at 66 cm from the X-ray tube). The proportion of fluoroscopy in the total dose was also calculated as the ratio of fluoroscopy DAP to total DAP.

### 2.3. Statistical Analysis

Statistical analyses were performed using the 3.5.1 version of R (R Core Team (2017); R: A language and environment for statistical computing; R Foundation for Statistical Computing, Vienna, Austria). For all quantitative data, normality was tested using the Shapiro–Wilk test. Data are presented as means and standard deviations or medians and 1st and 3rd quartiles, according to the variable statistical distribution. 

The comparison of dosimetric indicators between the A1 and A2 systems was performed for the following embolization procedures: BE, AEE, AUE, RE, UEE, UUE and GVE, and for HCE, PE and RaE for the comparison between the A1 and A3 systems. The comparison between all dosimetric indicators was performed using the paired Mann–Whitney–Wilcoxon test. A *p*-value less than 0.05 was considered significant.

## 3. Results

### 3.1. Patients

During the study period, 1449 procedures were performed. There were 472 women and 977 men, of mean age 59.9 ± 19.9 (SD) (range: 18.0–99.8) years old (Table 1). A total of 1089 embolization procedures of all 10 procedure types were performed with the A1 system, 222 embolization procedures were performed with the A2 system, including 7 types of procedures (BE, AEE, AUE, RE, UEE, UUE and GVE), and 138 procedures of 3 different types (HCE, PE, and RaE) were performed with the A3 system. No adult patients objected to their participation in the study but 26 pediatric patients were excluded.

### 3.2. Comparison of the Dosimetric Indicators between the Allura FD 20 (A1) and the Azurion 7 M20 (A2) Systems

**Figure 1:**
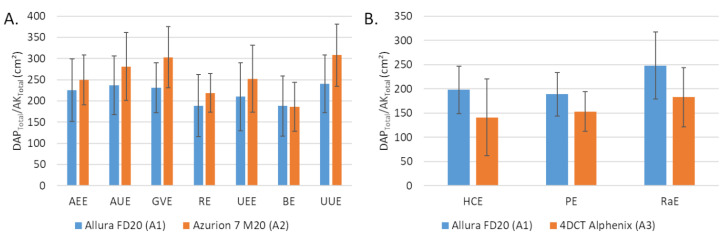
Average field size corresponding to the total dose area product (DAP) on total air kerma at 66 cm ratio (**A**) between Allura FD20 (A1) and Azurion 7 M20 (A2) for 7 embolization procedures and (**B**) between Allura FD20 (A1) and 4DCT Alphenix (A3) for 3 embolization procedures (**B**). Values are expressed as means ± standard deviations (error bars). DAP: dose area product; AK: air kerma; AEE: abdominal elective embolizations for tumors and visceral aneurysms; AUE: abdominal urgent embolizations for active bleedings or vascular injuries of digestive arteries; BE: bronchial artery embolizations; HCE: hepatic chemoembolizations; PE: pelvic embolizations for planned prostatic embolizations; RaE: radioembolisations; RE: renal artery embolizations; UEE: uterine elective embolizations for leiomyomas or vascular malformations; UUE: uterine urgent embolizations for postpartum hemorrhages; GVE: gonadal vein embolization.

The dosimetric indicator values obtained with the A1 and A2 systems for the 7 embolization procedures performed with A2 are depicted in Table 2. The DAP values were significantly lower with A2 compared to A1 for all procedures (*p* < 0.05), except for GVE and UEE. For these two procedures, the differences between the medians were −32% for GVE (*p* = 0.09) and −26% for UEE (*p* = 0.481) while they were on average of −50% ± 5% for the 5 other procedures. AK values were lower with A2 compared to A1 for all procedures (*p* < 0.05), except for UUE (*p* = 0.224). The average difference between the medians for the 6 procedures were −56% ± 8% but −23% for UEE. The average field size was higher with A2 than with A1 (Figure 1A).

For the fluoroscopy time, similar values were found between the A1 and A2 systems for AEE, AUE and RE. FT were higher with A2 compared to A1 for the other 4 procedures with significant differences for GVE (*p* < 0.001) and UEE (*p* = 0.001). 

**Figure 2:**
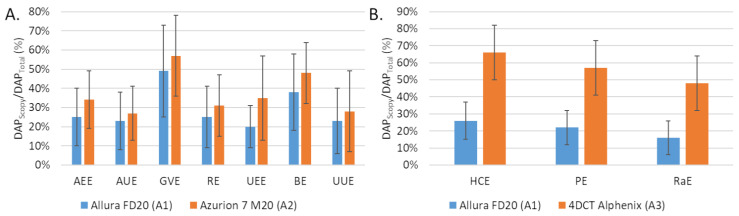
Comparison of the fluoroscopy dose area product (DAP) to total DAP ratio (**A**) between the Allura FD20 (A1) and Azurion 7 M20 (A2) systems for 7 embolization procedures and (**B**) between the Allura FD20 (A2) and 4DCT Alphenix (A3) systems for 3 embolization procedures. Values are expressed as means ± standard deviations (error bars). DAP: dose area product; AEE: abdominal elective embolizations for tumors and visceral aneurysms; AUE: abdominal urgent embolizations for active bleedings or vascular injuries of digestive arteries; BE: bronchial artery embolizations; HCE: hepatic chemoembolizations; PE: pelvic embolizations for planned prostatic embolizations; RaE: radioembolisations; RE: renal artery embolizations; UEE: uterine elective embolizations for leiomyomas or vascular malformations; UUE: uterine urgent embolizations for postpartum hemorrhages; GVE: gonadal vein embolization.

The number of fluorographies was significantly lower with A2 compared to A1 for AEE, AUE and RE but the opposite for the other embolization procedures (GVE, UEE, BE and UUE). The differences were significant for UEE (*p* = 0.022) and UUE (*p* = 0.032). The proportion scopy DAP in the total DAP was greater with A2 than with A1 (Figure 2A).

### 3.3. Comparison of the Dosimetric Indicators between the Allura FD20 (A1) and the 4DCT Alphenix (A3) Systems

The dosimetric indicator values obtained with the A1 and A3 systems for HCE, PE and RaE procedures are depicted in Table 3. The DAP values were significantly lower with A3 than with A1 (*p* < 0.001). The corrected AK with the A3 system were significantly lower than with A1 for PE and RaE (*p* < 0.001) but the opposite for HCE (*p* = 0.827). The average field size was lower with A3 compared with A1, 120 ± 67 cm² vs. 198 ± 49 cm² for HCE, 130 ± 35 cm² vs. 189 ± 45 cm² for PE, 156 ± 52 cm² vs. 248 ± 69 cm² for RaE (Figure 1B).

The fluoroscopy time was higher with A3 than with A1 for the 3 procedures and the differences were significant for HCE (*p* < 0.001) and PE (*p* = 0.024). The number of graphy images was significantly lower with A3 than with A1 for all procedures. The proportion was higher for A2 than for A1 (Figure 1B).

With the A3 system, the CT-scan was used for 91% of HCE and PE procedures and 88% for RaE (Table 4). The median DLP were 267 (185; 629) mGy.cm for HCE, 228 (139; 392) mGy.cm for PE and 282 (189; 530) mGy.cm for RaE. The median number of CT acquisitions were 3 (2; 4) for HCE, 3 (2; 3) for PE and 2 (2; 4) for RaE. 

**Figure 3:**
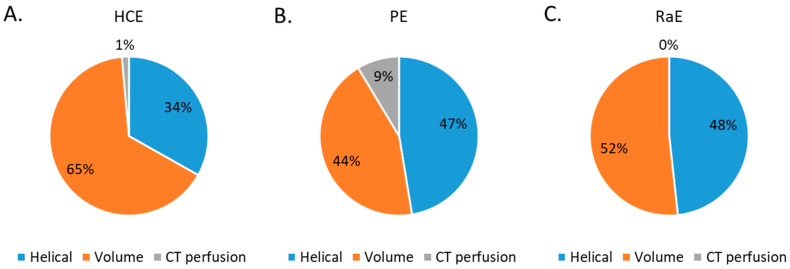
Percentage of CT acquisition types for all 3 embolization procedures. (**A**) HCE: hepatic chemoembolizations; (**B**) PE: pelvic embolizations for planned prostatic embolizations; (**C**) RaE: radioembolisations.

The proportion of CT acquisition types for the three embolization procedures studied are depicted in Figure 3. Volumic CT acquisitions represented 65% of the total CT acquisitions for HCE, 44% for PE and 52% for RaE. Perfusion CT acquisitions were used for HCE (1%) and PE (9%).

### 3.4. Comparison of the Dosimetric Indicators with the Litterature

The dosimetric indicator values for the 5 embolization procedures studied (BE, UEE, UUE, RE, HCE) were lower than the proposed DRL, except for the fluoroscopic time of BE with A2 and that of HCE with A3, and for the number of graph images of HCE with A3 (Table 5).

## 4. Discussion

A retrospective analysis of the doses delivered during 1449 thoracic, abdominal and/or pelvic embolization procedures was performed over a 10-year period during which three IR systems were used. The doses delivered in an older and a newer version of the IR system were compared. The contribution of CT in a multimodal room equipped with a scanner and a fixed C-arm was also compared to the older IR system.

The results of this study showed that the dosimetric indicator values collected for 7 embolization procedures were lower with the Azurion 7 M20 system (newer version) than with the Allura FD20 system (older version). AK values measured at the same interventional reference point were lower for the 7 embolization procedures studied. These differences were not related to differences in additional filtration or cadence for scopy (pulses/s) and graphy (images/s) as the same values were used with the two IR systems. They were directly related to the use of the ClarityIQ technology available in the new IR system. This technology was shown to reduce the image noise that improves image quality, and therefore reduces the dose while keeping the same image quality [16,17,18,19,20]. In this study, we found that the ClarityIQ technology reduced the AK by an average of 52% (24–65%), and the DAP to a lesser extent. Similar outcomes were found for AK reductions in different studies on uterine fibroid embolizations [16,18,19], bronchial artery embolization [20] and transarterial chemoembolization [17]. However, the reductions in DAP were smaller than those found in these studies [16,17,18,19,20]. This can be explained by the fact that the DAP variation was related to the increase in mean field size values, which may be related to the new service organization replacing a single room by two newly equipped rooms. Indeed, the room with the C-arm alone, which was mainly dedicated to the emergency embolizations and short-term endovascular procedures, was mostly used by junior radiologists (fellows and trainees). The complex and time-consuming procedures were usually performed in the 4DCT room by the senior radiologists. In the older IR room, the procedures were performed by both junior and senior radiologists. Junior radiologists often tend to use larger fields of exposure and increase the number of X-ray control, which resulted in an increased number of graphy images and a longer fluoroscopy time. This was reported for BE, UEE, UUE and GVE procedures while the other studies found similar or decreased fluoroscopy times using a C-arm system equipped with the ClarityIQ technology [16,17,18]. Training junior radiologists to good patient radiation protection practices is therefore essential to harmonize and improve practices, and thus reduce the doses delivered to the patients. Furthermore, the DAP and AK values obtained in this study for these two IR systems were lower than the national DRL values [10], which shows that the practices in our institution were already optimized [14] and were even more so with the arrival of this new IR system. Last, the median DAP values of UEE and BE for both C-arm systems used in our study were lower than the DAP values found in the literature [16,18,19,20]. For these procedures, our fluoroscopy times were within the range of those found in these studies [16,18,20]. 

The variations in dosimetric indicators obtained between the older IR system and the 4DCT room depended on the procedure performed. The AK values were lower with the 4DCT than with the older IR system for PE and RaE procedures but for HCE AK values were slightly higher with the 4DCT. These results are directly related to the use of CT acquisitions during the procedure, which changes patient management. For PE and RaE procedures, the helical and volume angiographic CT acquisitions were performed at the beginning, during and at the end of the procedure. These images provide a more accurate anatomy and a 3D artery volume, which could be merged with the IR images to improve treatment planning and simplify the procedure. Conversely, for HCE procedures, the use of CT angiography allows the treatment of several targets that could not be treated with standard IR systems and CBCT acquisitions. An ancillary study could be carried out to correlate the AK values with the number of targets treated. Additionally, the use of CT during the procedure changed the operators’ practices. Compared to the older IR system, the fluoroscopy times were increased but the number of graphy images were significantly reduced. The radiologists do their planning under CT and their follow-up with fluoroscopy with a sufficient and adapted image quality. This reduces the radiologist’s need for digital subtraction angiography acquisitions, which significantly reduces the number of graphy images. It should also be noted that the average field size was reduced using the Alphenix C-arm. In contrast to Azurion 7 M20, the most complex procedures were performed in the 4DCT system with the help of CT and were performed by senior radiologists who were more aware of the good practices. The AK and DAP values obtained for HCE were lower than the national DRL values [10]. However, the DAP, AK and fluoroscopy time values were higher in our study than those found by Piron et al. [15] for HCE. Although this result may be explained by the differences in the complexity of the procedures and the number of targets treated (not evaluated in this study), optimization of the procedure is required to be implemented in our 4DCT system to reduce the dose delivered to patients. Additionally, the dose reduction tools proposed by Canon “Live Zoom” and “Spot fluoro” were rarely used in the 4DCT room whereas they were used for all HCE procedures in the Piron et al. study [15]. Awareness of the use of these tools in the 4DCT room should be performed by the interventional radiologists. On the other hand, the DLP values found in our study were lower than those found by Piron et al. [15]. This result may be linked to the use of a deep-learning image reconstruction algorithm (AiCE) in our CT system compared to the iterative reconstruction algorithm (AIDR 3D) used in their study [24]. Indeed, this new algorithm was shown to improve the image quality and have a high potential for dose reduction compared to the iterative reconstruction algorithm.

This study has some limitations. It reflects the practices of a single center, with a 10-year experience of thorough optimization processes and the presence of two medical physicists. As the two new IR systems were installed in September 2020, the patient samples may be different from the older IR system. However, the number of patients was sufficient to perform a statistical analysis. In addition, this study only focused on the dosimetric indicators; clinical factors were not taken into account. Another limitation is that the experience of the operators was only indirectly taken into account in the study of the new organization with two new rooms dedicated to the junior and senior radiologists, unlike the older room. The 4DCT, with its much higher anatomical precision, allows carrying out more complex procedures which should impact the dose received by the patients [25]. Furthermore, we did not evaluate the differences in image quality between the different rooms for the different procedures studied. Also, the acquisition protocols were defined by the medical physicist and the application engineer and the resulting image quality was validated by the interventional radiologists in each room. However, for some patients and some complex procedures, the image quality proposed may not have been sufficient and adapted, especially in scopy, which may explain the higher scopy time in the 4DCT room. Further targeted studies will be carried out to validate the image quality. Last, ancillary studies may now be performed to correlate the dose indicators with the number of targets or with the type of arteries/veins or organs treated.

## 5. Conclusions

This monocentric retrospective analysis of the doses delivered during thoracic, abdominal and pelvic embolization procedures over a 10-year period showed the contribution of the new IR tools in dose reduction and patient management. The last C-arm technology reduced the image noise and improved image quality, allowing a 50% reduction of the air kerma and showing a significant dose reduction. The implementation of a CT scan inside the IR room allowed a more precise 3D guidance without increasing the dose delivered to the patients.

## Figures and Tables

**Table 1:** Patients’ characteristics.

	Allura FD 20 (A1)				Azurion 7 M20 (A2)		Alphenix 4DCT (A3)		
Procedures	Number of	Sex	Age	Number of	Sex	Age	Number of	Sex	Age
	Patients	(F/M)	(Years)	Patients	(F/M)	(Years)	Patients	(F/M)	(Years)
AEE	203	58/145	60.4 ± 18.9	58	19/39	64.5 ± 17.7	-	-	-
AUE	123	38/85	66.5 ± 16.4	38	16/22	64.5 ± 17.3	-	-	-
GVE	156	32/124	36.4 ± 13.3	30	13/17	40.7 ± 13.1	-	-	-
RE	99	50/49	63.4 ± 18.0	25	6/19	67.7 ± 15.8	-	-	-
UEE	81	81/0	41.2 ± 12.4	18	18/0	46.9 ± 16.5	-	-	-
BE	44	9/35	61.9 ± 16.2	36	10/26	64.2 ± 15.9	-	-	-
UUE	60	60/0	31.8 ± 6.2	17	17/0	31.4 ± 7.8	-	-	-
HCE	158	22/136	70.3 ± 9.8	-	-	-	67	12/55	72.2 ± 9.6
PE	142	0/142	76.5 ± 10.1	-	-	-	47	0/47	76.5 ± 12.1
RaE	23	1/22	70.6 ± 6.8	-	-	-	24	10/14	66.8 ± 10.9
Total	1089	351/738	58.7 ± 20.2	222	99/123	57.6 ± 19.7	138	22/116	72.7 ± 11.2

Age values are expressed as means ± standard deviations. AEE: abdominal elective embolizations for tumors and visceral aneurysms; AUE: abdominal urgent embolizations for active bleeding or vascular injuries of digestive arteries; BE: bronchial artery embolizations; HCE: hepatic chemoembolizatiosn; PE: pelvic embolizations for planned prostatic embolizations; RaE: radioembolisations; RE: renal artery embolizations; UEE: uterine elective embolizations for leiomyomas or vascular malformations; UUE: uterine urgent embolizations for postpartum hemorrhages; GVE: gonadal vein embolizations.

**Table 2:** Comparison of the dosimetric indicators between the Allura FD20 (A1) and Azurion 7 M20 (A2) C-arms systems for 7 embolization procedures.

Procedures	Dosimetric Indicators	Allura FD20 (A1)	Azurion 7 M20 (A2)	*p*-Values
AEE	Dose Area Product (Gy.cm^2^)	110.9 (54.6; 186.4)	48 (29.3; 85.9)	***p < 0.001***
	Air Kerma (mGy)	510 (264; 959)	207 (130; 337)	***p < 0.001***
	Fluoroscopy Time (min)	17 (11; 28)	17 (11; 23)	0.676
	Number of graphy images	133 (84; 246)	109 (70; 190)	0.063
AUE	Dose Area Product (Gy.cm^2^)	126.0 (65.2; 238.9)	61.3 (36.5; 103.4)	***p < 0.001***
	Air Kerma (mGy)	566 (278; 1097)	216 (132; 375)	***p < 0.001***
	Fluoroscopy Time (min)	19 (9; 29)	17 (12; 27)	0.550
	Number of graphy images	157 (93; 330)	148 (88; 216)	0.316
GVE	Dose Area Product (Gy.cm^2^)	31.4 (20.1; 63.2)	21.5 (14.3; 52.5)	0.09
	Air Kerma (mGy)	141 (87; 258)	66 (36; 105)	***p < 0.001***
	Fluoroscopy Time (min)	16 (11; 24)	29 (20; 38)	***p < 0.001***
	Number of graphy images	41 (22; 77)	66 (35; 107)	0.153
RE	Dose Area Product (Gy.cm^2^)	83.8 (41.7; 125.2)	42.8 (35.5; 98.3)	***0.019***
	Air Kerma (mGy)	461 (273; 808)	207 (148; 381)	***0.001***
	Fluoroscopy Time (min)	17 (11; 25)	16 (8; 22)	0.542
	Number of graphy images	151 (91; 223)	105 (73; 182)	0.166
UEE	Dose Area Product (Gy.cm^2^)	92.1 (49; 161.9)	68.1 (35.5; 158.6)	0.481
	Air Kerma (mGy)	417 (239; 862)	319 (189; 661)	0.224
	Fluoroscopy Time (min)	17 (11; 27)	30 (20; 43)	***0.001***
	Number of graphy images	112 (79; 208)	186 (133; 313)	***0.022***
BE	Dose Area Product (Gy.cm^2^)	49.4 (29.9; 79.6)	29.7 (20.5; 47.3)	***0.010***
	Air Kerma (mGy)	288 (148; 478)	166 (114; 235)	***0.012***
	Fluoroscopy Time (min)	24 (17; 40)	31 (21; 43)	0.319
	Number of graphy images	131 (93; 196)	128 (101; 252)	0.315
UUE	Dose Area Product (Gy.cm^2^)	166.9 (86.3; 273.4)	80.3 (51.8; 130.4)	***0.009***
	Air Kerma (mGy)	710 (423; 1092)	249 (161; 497)	***0.002***
	Fluoroscopy Time (min)	14 (9; 22)	18 (9; 24)	0.815
	Number of graphy images	108 (59; 182)	175 (121; 260)	***0.032***

*p*-values in bold italics are significant *p*-values (<0.05). AEE: abdominal elective embolizations for tumors and visceral aneurysms; AUE: abdominal urgent embolizations for active bleedings or vascular injuries of digestive arteries; BE: bronchial artery embolizations; RE: renal artery embolizations; UEE: uterine elective embolizations for leiomyomas or vascular malformations; UUE: uterine urgent embolizations for postpartum hemorrhages; GVE: gonadal vein embolization.

**Table 3:** Comparison of the dosimetric indicators between the Allura FD20 (A1) and 4DCT Alphenix (A3) C-arms systems for 3 embolization procedures.

Procedures	Dosimetric Indicators	Allura FD20 (A1)	Alphenix 4DCT (A3)	*p*-Values
HCE	Dose Area Product (Gy.cm^2^)	126.9 (72.7; 188.6)	91.4 (44; 127.5)	***p < 0.001***
	Air Kerma (mGy)	672 (365; 1074)	***699 (391; 1005)***	0.827
	Fluoroscopy Time (min)	24 (16; 34)	37 (26; 48)	***p < 0.001***
	Number of graphy images	403 (144; 760)	80 (58; 139)	***p < 0.001***
	Dose Length Product (mGy.cm) (*n* = 61)	-	267 (185; 629)	-
PE	Dose Area Product (Gy.cm^2^)	239.1 (135.1; 322.7)	134.1 (96.2; 161.7)	***p < 0.001***
	Air Kerma (mGy)	1314 (735; 1849)	***874 (629; 1306)***	***0.012***
	Fluoroscopy Time (min)	43 (30; 54)	50 (40; 60)	***0.024***
	Number of graphy images	1018 (638; 1345)	191 (155; 280)	***p < 0.001***
	Dose Length Product (mGy.cm) (*n* = 43)	-	228 (139; 392)	-
RaE	Dose Area Product (Gy.cm^2^)	103.4 (78; 156.4)	36.6 (19.1; 56.1)	***p < 0.001***
	Air Kerma (mGy)	498 (305; 657)	***258 (130; 319)***	*0.001*
	Fluoroscopy Time (min)	11 (8; 20)	20 (12; 25)	0.140
	Number of graphy images	783 (662; 1067)	83 (48; 114)	***p < 0.001***
	Dose Length Product (mGy.cm) (*n* = 21)	-	282 (189; 530)	-

HCE: Hepatic chemoembolizations; PE: Pelvic embolizations for planned prostatic embolizations; RaE: Radioembolisations.

**Table 4:** Dose length product and number of CT acquisitions.

Dosimetric Indicators	HCE	PE	RaE
Number of embolizations	67	47	24
Number of procedures	61	43	21
Dose Length Product (mGy.cm)	267 (185; 629)	228 (139; 392)	282 (189; 530)
Number of CT acquisitions	3 (2; 4)	3 (2; 3)	2 (2; 4)

HCE: hepatic chemoembolizations; PE: pelvic embolizations for planned prostatic embolizations; RaE: radioembolisations.

**Table 5:** Comparison of dosimetric indicators with the reference levels proposed by Etard et al. [10].

Dosimetric Indicators	Systems	BE	UEE	UUE	RE	HCE
Dose Area Product (Gy.cm^2^)	Allura FD 20 (A1)	49	92	167	84	127
	Azurion 7 M20 (A2)	30	68	80	43	-
	4DCT Alphenix (A3)	-	-	-	-	91
	DRL	135	175	255	325	250
Air Kerma (mGy)	Allura FD 20 (A1)	288	417	710	461	672
	Azurion 7 M20 (A2)	166	319	249	207	-
	4DCT Alphenix (A3)	-	-	-	-	699
	DRL	830	800	930	1700	990
Fluoroscopy time (min)	Allura FD 20 (A1)	24	17	14	17	24
	Azurion 7 M20 (A2)	31	30	18	16	-
	4DCT Alphenix (A3)	-	-	-	-	37
	DRL	38	29	25	22	28
Number of graphy images	Allura FD 20 (A1)	131	112	108	151	403
	Azurion 7 M20 (A2)	128	186	175	105	-
	4DCT Alphenix (A3)	-	-	-	-	80
	DRL	240	160	260	210	200

BE: bronchial artery embolizations; RE: renal artery embolizations; UEE: uterine embolizations for leiomyomas or vascular malformations; UUE: uterine urgent embolizations for postpartum hemorrhages; HCE: hepatic chemoembolizations. DRL: dose reference levels proposed by Etard et al. corresponding to the third quartile rounded to the nearest integer [10].

## Data Availability

The data presented in this study are available on request from the corresponding author.
